# CoSpliceNet: a framework for co-splicing network inference from transcriptomics data

**DOI:** 10.1186/s12864-016-3172-6

**Published:** 2016-10-28

**Authors:** Delasa Aghamirzaie, Eva Collakova, Song Li, Ruth Grene

**Affiliations:** 1 Genetics, Bioinformatics and Computational Biology, Virginia Tech, Blacksburg, VA 24061 USA; 2 Department of Plant Pathology, Physiology, and Weed Science, Virginia Tech, Blacksburg, VA 24061 USA; 3 Department of Crop and Soil Environmental Sciences, Virginia Tech, Blacksburg, VA 24061 USA

## Abstract

**Background:**

Alternative splicing has been proposed to increase transcript diversity and protein plasticity in eukaryotic organisms, but the extent to which this is the case is currently unclear, especially with regard to the diversification of molecular function. Eukaryotic splicing involves complex interactions of splicing factors and their targets. Inference of co-splicing networks capturing these types of interactions is important for understanding this crucial, highly regulated post-transcriptional process at the systems level.

**Results:**

First, several transcript and protein attributes, including coding potential of transcripts and differences in functional domains of proteins, were compared between splice variants and protein isoforms to assess transcript and protein diversity in a biological system. Alternative splicing was shown to increase transcript and function-related protein diversity in developing Arabidopsis embryos. Second, CoSpliceNet, which integrates co-expression and motif discovery at splicing regulatory regions to infer co-splicing networks, was developed. CoSpliceNet was applied to temporal RNA sequencing data to identify candidate regulators of splicing events and predict RNA-binding motifs, some of which are supported by prior experimental evidence. Analysis of inferred splicing factor targets revealed an unexpected role for the unfolded protein response in embryo development.

**Conclusions:**

The methods presented here can be used in any biological system to assess transcript diversity and protein plasticity and to predict candidate regulators, their targets, and RNA-binding motifs for splicing factors. CoSpliceNet is freely available at http://delasa.github.io/co-spliceNet/.

**Electronic supplementary material:**

The online version of this article (doi:10.1186/s12864-016-3172-6) contains supplementary material, which is available to authorized users.

## Background

Alternative splicing (AS) is a ubiquitous phenomenon occurring across all eukaryotic organisms as many biological processes are regulated through this type of post-transcriptional process, leading to the production of more than one coding or noncoding transcript from a single locus [1–3]. Several types of AS events occur during precursor mRNA (pre-mRNA) splicing, including exon skipping, intron retention, and/or the use of alternative acceptor and donor splice sites, producing transcripts with premature stop codons and altered coding potential [4]. AS provides a basis for protein diversity by generating proteins with distinct amino acid sequences, leading to altered numbers and types of functional domains, protein modification sites, or truncated proteins with different biological functions [5–7]. Because many of these protein isoforms are membrane bound, not abundant, and often too short to be captured by proteomics approaches [8–10], alternative approaches are needed to assess the influence of AS on protein diversity.

Pre-mRNA splicing involves over 150 regulatory spliceosomal components, including small ribonucleoproteins, specific splicing factors (SFs), and other proteins, collectively referred to as splicing-related proteins (SRPs) [11]. SRPs are involved in protein-RNA (RNA-binding proteins (RBPs) such as SFs) and/or protein-protein interactions within a spliceosome. The final splicing outcome is the result of the action of several SRPs. The specificity of splicing is brought about through the action of SFs, which bind to their target pre-mRNAs in a position-dependent and RNA-motif-specific manner, acting as exonic or intronic splicing enhancers or silencers [12]. The position of splicing regulatory elements determines the action of the cognate SF because they affect the representation or misrepresentation of the splice site to the SF, which ultimately results in inclusion or exclusion of the corresponding RNA sequence in the final transcript [12]. This being the case, the production of spliced transcripts is dependent, in part, on the presence and activity of each SF required for the splicing of its corresponding pre-mRNAs. Coordination exists between the expression of an SF and the transcripts produced by that SF. Coordinated splicing (co-splicing) is defined here as the action of the spliceosome on a group of pre-mRNAs to produce a population of coordinately expressed and spliced transcripts.

Extensive protein-RNA binding information based on Photoactivatable Ribonucleoside-Enhanced Crosslinking and Immunoprecipitation (PAR-CLIP) [13] and CLIP-seq-based [14] experiments is only available in animals. In human and mouse, several computational tools have been developed to integrate protein-RNA binding data with splicing patterns to define the splicing code [15], including tissue-specific splicing code [16], and to infer co-splicing networks for specific regulatory SFs (e.g., NOVA [17]). Computational pipelines have been implemented to identify conserved RNA-binding motifs for individual RBPs using these types of data [18, 19]. Since direct protein-RNA binding data is lacking for other organisms [4, 20], computational tools are needed that can systematically identify putative SFs, predict RNA-binding sites for the corresponding pre-mRNA targets of SFs of interest, and infer global networks of co-spliced product transcripts.

Here, we introduce CoSpliceNet, an integrated computational framework for unraveling co-splicing regulation on a global scale using RNA sequencing (RNA-Seq) data and *de novo* predictions of SF RNA-binding sites. The CoSpliceNet framework was applied to existing temporal and organ-specific co-expression data obtained from developing *Arabidopsis thaliana* embryos [21] to infer a co-splicing network for 13 selected RBPs that are differentially expressed during embryo development. The tool can be easily applied to any temporal or other RNA-Seq or splicing microarray datasets obtained from any eukaryotic organism to infer predictive co-splicing networks.

## Results

### AS and protein diversity in Arabidopsis embryo development

**Fig. 1:**
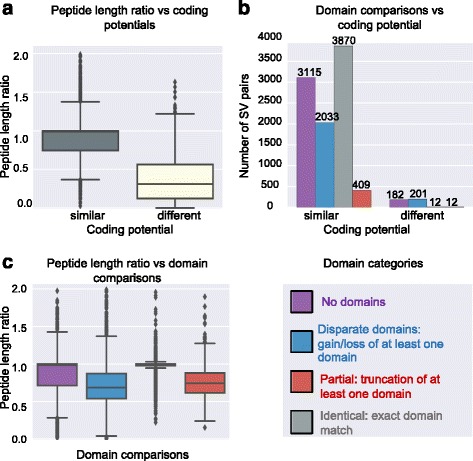
Protein diversity assessment of transcripts expressed during Arabidopsis embryo development. Five thousand six hundred two genes were alternatively spliced. Protein diversity analysis was performed on 9834 SV pairs of these genes. **a** Effect of coding potential on peptide length differences of protein isoforms. **b** Relationship between the domain composition and coding potential. **c** Relationship between peptide length ratio and domain composition of protein isoforms.

In order to characterize the effect of AS on protein diversity, first, genes encoding pre-mRNAs that were alternatively spliced were identified. Five thousand six hundred two genes were identified that were alternatively spliced from the total population of 53,988 detected transcripts. These genes encoded at least one non-canonical SV (9834 transcripts in total, see Table 1 for definition of canonical SV) each, which was compared in each case with the canonical SV for protein diversity analysis. Assessment of differences in coding potentials, peptide ratios, pairwise global alignment scores, and functional domain compositions were carried out (see Methods). Among these 9834 SV pairs, only 407 genes produced both a coding and a noncoding SV. In cases when a SV was predicted to be noncoding, the corresponding “peptide length” ratio was found to be 0.25 on average (Fig. 1a) as non-coding RNAs typically contain short open reading frames [27].

**Table 1:** Terminology and the corresponding definitions used in this manuscript.

Term	Definition
Canonical transcript	The splice variant with the lowest isoform number among known transcripts in the current database (e.g. TAIR10). For example, if gene X has two known transcripts X.1 and X.2, as specified in the database, X.1 is defined as the canonical form
Co-spliced transcripts	The transcripts containing common RNA-binding motifs that are co-expressed with a specific SF
Differentially spliced	Splice variants transcribed from the same gene that are spliced by different SFs.
Peptide ratio	The length ratio of a given non-canonical protein isoform to the canonical protein isoform
Protein isoforms	The proteins that are synthesized from different splice variants
Ri region (1 ≤ i ≤ 4)	R1 (-31:-1 5′ss), R2 (0:30 5′ss), R3 (-30:0 3′ss), and R4 (1:31 3′ss) sequences for each exon in a transcript
Ri ratio	The ratio of the number of exons containing a motif in Ri region to the total number of exons
Splice variant (SV)	Transcripts that are products of the same precursor mRNA.
Splicing-related proteins (SRPs)	Proteins known to be involved in the spliceosome machinery
Splicing factor (SF)	SRPs with known RNA-binding domains
Super-cluster	Clusters of transcripts with similar expression profiles grouped according to known Arabidopsis seed developmental stages.

Our results revealed that AS may result in the production of protein isoforms with identical, disparate, or truncated domains. Among the 9834 SV pairs, about 40 % (3870) of inferred protein isoforms contained identical domains, indicating that AS affected amino acid sequences other than those in the conserved domains. Approximately 23 % of protein isoforms (2234 out of 9834 SV pairs) were missing a domain completely (disparate domains). About 4.2 % (421 out of 9834 SV pairs) of the protein isoforms had truncated domains, suggesting that AS partially affected functional domains as opposed to removing them altogether (Fig. 1b). The majority of protein isoforms originating from transcripts with similar coding potentials had either identical or similar domains (3870 and 3115 respectively, Fig. 1b). Comparing conserved domain differences with the population of protein isoform lengths revealed that short protein isoforms had either truncated domains or had lost a domain completely (Fig. 1c). Therefore, AS events that result in the production of a protein isoform that is approximately 30 % shorter than the canonical protein isoform have a higher probability of causing loss or truncation of the functional domains (Fig. 1c).

Because a main focus on this manuscript is on the differentially expressed transcripts, we performed protein diversity analysis on them as well. Two thousand three hundred forty-five genes were identified that were alternatively spliced. Each non-canonical SV was compared with its corresponding canonical SV, leading to the identification of 3008 SV pairs (a gene can have more than one non-canonical SV). These 3008 SV pairs were subjected to protein diversity analysis. The differentially expressed transcripts follow the same distributions as the whole population of detected transcripts in the protein diversity categories (Additional file 1: Figure S1).

### Characterization of differentially expressed transcripts in developing Arabidopsis embryos

Identification of the set of transcripts whose expression changed significantly in developing Arabidopsis embryos is of central importance for understanding any time and/or developmentally dependent relationships that may exist between the action of specific SFs and their targets. Therefore, further analysis for co-splicing network inference was performed on this specific set of differentially expressed transcripts. The population of 7960 differentially expressed transcripts was categorized into coding or noncoding based on CodeWise [7] predictions and genic or intergenic, sense or antisense, coding or non-coding as defined in the “Tuxedo Suite” package [7, 25, 26] (Additional file 2: Table S1). Most differentially expressed transcripts were known, or predicted, to be coding, with only 429 differentially expressed ncRNAs detected in the developing Arabidopsis embryo dataset (Table 2).

**Table 2:** Categorization of 7960 differentially expressed transcripts into Cuffcompare classes. Differentially expressed transcripts in developing Arabidopsis embryos belong to different classes (known, novel splice junction, exon skipping, antisense, and intergenic) and can be coding or noncoding based on CodeWise predictions.

Transcripts (Cuffcompare class)	Coding	Noncoding
Known (=)	5990	144
Novel splice junction (j)	1474	70
Exon skipping (o)	62	25
Antisense (x and s)	3	124
Intergenic	2	66
Total	7531	429

### Co-expression network analysis

**Fig. 2:**
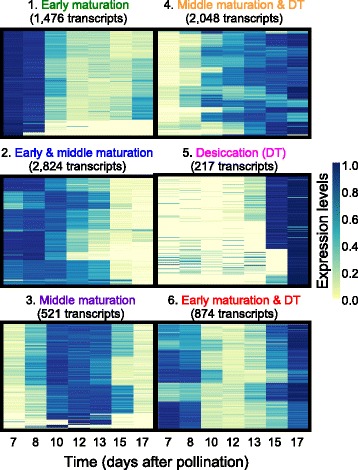
Classification of transcripts into super-clusters. The set of 7960 differentially expressed transcripts were grouped into 50 clusters using k-means clustering. Clusters were further merged into 6 super-clusters based on the extent of transcript expression rather than the actual expression profiles during the three major developmental phases in Arabidopsis seed maturation (early and middle maturation, and early desiccation). The colors assigned to each super-cluster were used to visualize the nodes (transcripts) in all networks within this manuscript to obtain temporal information on transcript expression in developing Arabidopsis embryos.

To identify trends among 7960 differentially expressed transcripts, k-means clustering was performed to obtain 50 clusters (Additional file 3: Figure S2). Grouping of clusters containing transcripts that were expressed at the same developmental phase yielded six super-clusters, containing six combinations of the three embryo maturation phases defined above (Fig. 2). These super-clusters comprised transcripts expressed at: (i) early maturation, (ii) early and middle maturation, (iii) middle maturation, (iv) middle maturation and early desiccation, (v) early desiccation, and (vi) both early maturation and desiccation phases. Grouping transcripts into color-coded super-clusters facilitated visualization of co-expression and co-splicing networks from the temporal perspective of embryo development. Although some transcripts were expressed only at one developmental phase, the expression of the majority of differentially expressed transcripts (~73 %) spanned two or more developmental phases (Fig. 2).

**Fig. 3:**
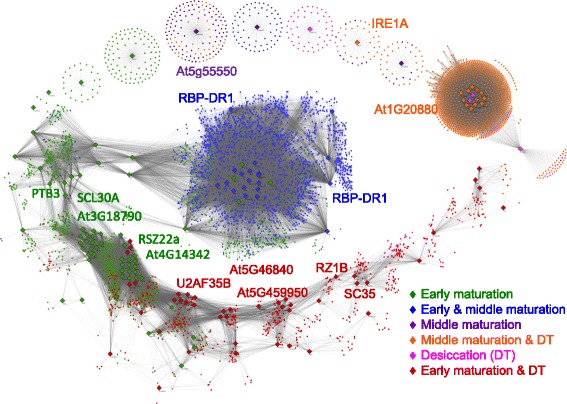
Association of differentially expressed transcripts with SRPs in a co-expression network. Spearman correlation analysis was performed between 146 SRPs and 7960 differentially expressed transcripts. Transcripts showing temporal trends that highly correlated with each SRP (gray edges: Spearman correlation coefficient > 0.95) were extracted (6341 transcripts) and visualized as a co-expression network in Cytoscape. SRPs are shown as diamonds and transcripts as circles. Nodes are color-coded based on super-clusters introduced in Fig. 2. The 14 RBPs are presented.

The set of 7960 significantly differentially expressed transcripts contained 146 SRPs. These SRPs were distributed across the six super-clusters (Additional file 4: Table S2), however, 42 % of SRPs were expressed during the “early” (62 out of 146) and 30 % of SRPs were expressed during “early maturation and desiccation” phases of embryo development (44 out of 146) (Additional file 5: Figure S3). To identify associations between SRPs and their potential products, Spearman correlation analysis was performed on the set of 146 SRPs and the set of 7814 remaining differentially expressed transcripts. This analysis led to the identification of 6341 transcripts whose expression was highly correlated with at least one SRP (*p*-value < 0.001, r > 0.95). A list of transcripts (and their properties) associated with each SRP can be found in Additional file 6: Table S3. The resulting co-expression network is shown in Fig. 3.

As shown in this network, some transcripts were co-expressed only with a single SRP, forming individual sub-networks (e.g., IRE1A or At5g55550). The majority of transcripts, however, co-expressed with more than one SRP, resulting in a highly interconnected large sub-network with potential associations among individual SRPs (e.g., the RBP-DR1, PAB7, and BIP sub-networks). Although most edges in this network likely reflect transcriptional co-expression, some may reflect co-splicing relationships, or a combination of transcriptional co-expression and co-splicing. To further explore possible specific splicing-related associations between SRPs and their product transcripts, co-splicing networks were constructed by integration of *de novo* motif discovery at the splice junctions.

### Co-splicing network inference by using CoSpliceNet

**Fig. 4:**
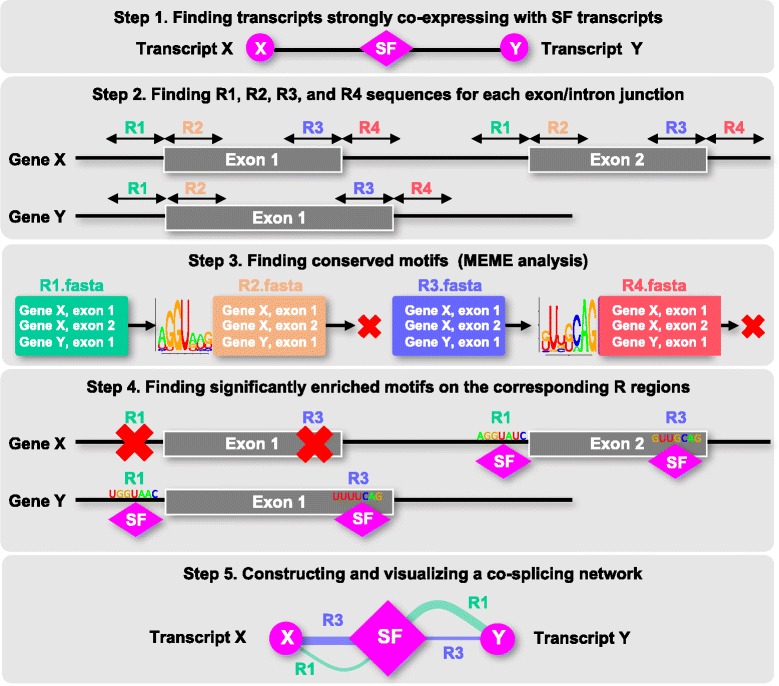
Co-splicing network construction. The following steps were performed to construct a co-splicing network: First, the sets of transcripts highly correlating with at least one SRP (Spearman correlation coefficient > 0.95) were identified. Second, 30-nucleotide regions surrounding each exon/intron splice junction (R1 through R4) were extracted for each transcript co-expressing with any of the selected RBPs. In this example, transcripts X and Y are encoded by genes X and Y. Third, the resulting sequences were subjected to MEME analysis separately for each of the SF groups to find consensus motifs. Fourth, transcripts containing significantly enriched motifs at each region (*p*-value < 0.05) were retrieved from the MEME results. Fifth, for each transcript that had a significantly enriched motif in at least one of the R regions (e.g., R1 and R3 for co-expressing genes X and Y), the corresponding weighted edges representing the individual R regions were constructed between each SF and its potential product in Cytoscape. Edges are weighted based on –log (*p*-value).

Because not every SRP is involved in pre-mRNA-protein interactions and the goal was to identify SRPs responsible for splicing specificity, the population of 146 SRPs was mined to identify genes encoding SFs and RBPs that possess potential RNA-binding capabilities based on experimental evidence and/or the presence of at least one single RNA-binding domain. This resulted in the identification of 14 transcripts encoding RBPs with an RNA-binding domain (Additional file 7: Table S4). Transcripts belonging to the sub-networks of these 14 RBPs were used for subsequent motif discovery and co-splicing network construction (Fig. 4). The transcripts whose expression was positively correlated with any of these 14 RBPs (2646 transcripts) were extracted from the large co-expression network (Additional file 8: Table S5). *De novo* motif discovery was performed using MEME [28] as described in Methods to identify consensus sequences in the splice junctions, specifically in the 30-nucleotide (R1 – 4) R-regions, of co-expressed transcripts with each RBP. R1 and R4 are within intronic, while R2 and R3 are within exonic regions of the splice junctions (Fig. 4). The transcripts that contained at least one significant motif (*p*-value was below 0.05 compared with background noise) at one of their R regions (R1 – 4) were retained for the co-splicing network analysis. The motif was then incorporated into the final predictive co-splicing network, such that an edge was formed between the RBP and its predicted products whose pre-mRNAs had that motif in the corresponding R region. Co-splicing networks were constructed only for RBPs to specifically predict connections between proteins capable of interacting with RNA and their products at each splice junction.

To construct a co-splicing network related to the population of 14 RBPs and their potential products, an edge was formed between transcripts co-expressing with any of the 14 RBPs when at least one conserved RNA motif was present in any of the four R regions, as defined above. The resulting co-splicing network contained 2074 transcripts connected through at least one R-related edge to at least one specific RBP (Additional file 9: Table S6). Please note that no significantly enriched motif was found for SCL30 and, as such, this network contained 13 RBPs. All R regions that did not have statistically significant motifs compared with background noise (*p*-value < 0.05) were eliminated from the co-splicing network (Additional file 10: Table S7). A transcript was predicted as a potential target of an SF if the expression of that transcript was highly correlated (*p*-value < 0.001, Spearman correlation coefficient > 0.95) with the expression of that SF in developing embryos and a statistically significantly occurring enriched motif existed in at least one of the R-regions (*p*-value < 0.05).

In order to assess how frequently the motifs occur in exon/intron junctions, the Ri ratio was defined (see Methods). We compared the sequence motifs detected in the R1 through R4 regions for each group and identified motifs that were specifically enriched in each region. The list of 13 RBPs and their corresponding significantly enriched motifs in each Ri region are available in Additional file 11: Table S8 in the format of motif logos and in text format in Additional file 12: Table S9. The position weight matrices for these motifs are available in Additional file 13: Table S10. Some motifs were found to be present in multiple co-splicing networks. For example, the AGGU motif was enriched in more than half of the R1 regions, and the UGCAG motif in most of the R4 regions. C/U-rich motifs were found in the majority of R2 and R3 regions with a consensus sequence of CUUCUU.

### Identification of differentially spliced transcripts

The networks presented above are based on transcriptional co-expression and/or co-splicing associations between SRPs and transcripts that showed expression trends nearly identical to the trends of these SRPs. The expression profile of a pre-mRNA is dependent on the action of specific TFs, but pre-mRNAs are transient and usually not captured by RNA-Seq data as most splicing co-occurs with transcription in the nucleus. The resulting transcripts can either show trends that are similar to those of their corresponding pre-mRNA (transcriptional), trends that correspond to the action of a SF (splicing), or a combination of the two. One way to computationally distinguish co-splicing from transcription-related co-expression is to identify SVs that show different expression profiles. SVs are encoded by the same gene and any differences in their expression profiles can be explained by a post-transcriptional event, e.g., differential splicing when distinct SFs bind to their motifs on pre-mRNA to facilitate splicing, in our case, SFs expressed at different developmental stages.

**Fig. 5:**
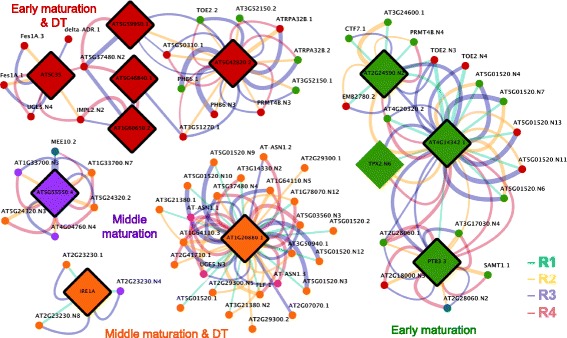
Co-splicing sub-networks for differentially expressed and differentially spliced transcripts. This sub-network shows transcripts represented by at least two SVs connected with different SRPs. This analysis separates transcriptional co-expression from co-splicing.

Differentially spliced transcripts were defined here as SVs of a gene encoding a pre-mRNA predicted to be spliced by distinct SFs (based on the constructed co-splicing network) belonging to different super-clusters. Fifty two genes were identified that encode differentially spliced transcripts (SVs), having at least one SV connected to one of the 13 RBPs and at least one other either differentially expressed SV or an SV that showed stable expression during embryo development (Additional file 14: Table S11). The resulting splicing-specific network contained 11 RBPs and 68 transcripts (Fig. 5). For example, the *TOE2* gene in this network has one SV (*TOE2.2*) associated with the RBP At5g42820.2 and two other SVs (*TOE2.N3* and *TOE2.N4*) were connected to the RBP At2g34590 and At4g14342. In contrast, *Fes1A.1* and *3* co-express with the SF SC35 (expressed during early maturation and desiccation phases), while the third SVs (*Fes1A.N4*) is only expressed during the seed desiccation phase and is not associated with any of the 11 RBPs within this sub-network.

### Inferred splicing events associated with the UPR in the ER

**Fig. 6:**
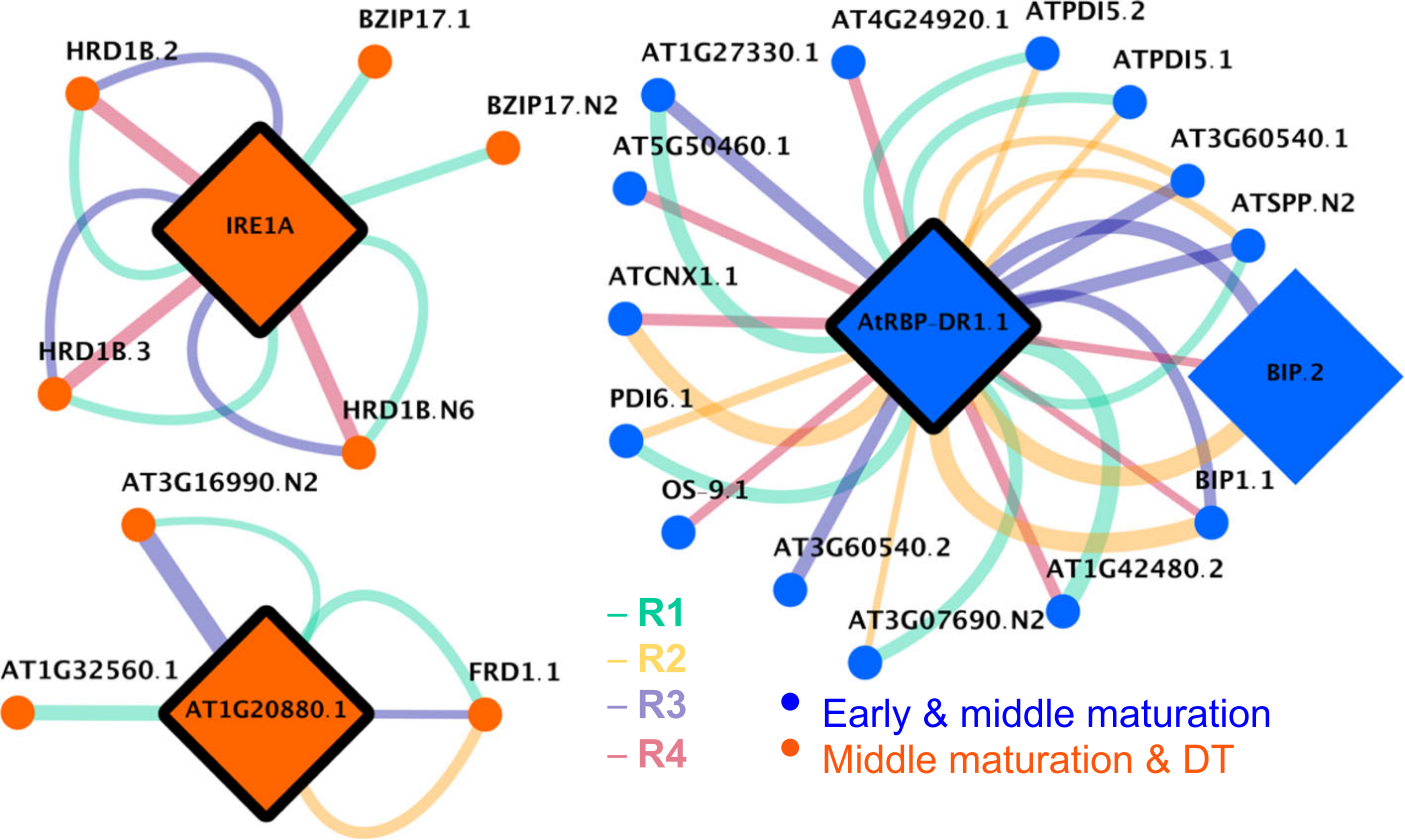
Co-splicing sub-networks for ER-related transcripts. IRE1A, AtRBP-DR1, and At1g20880 are RBPs involved in ER-associated splicing and are expressed during early and middle maturation or middle maturation and desiccation phases of embryo development. Associations between these RBPs and their predicted products are shown through weighted edges (the thickness of the edges corresponds to the confidence for the association), representing the presence of significantly enriched conserved motifs in the corresponding R regions of these products.

ER stress occurs in plants under specific conditions [35], one of which may be the intense production of secretory proteins during specific developmental phases. This is manifested as the accumulation of unfolded or misfolded proteins in the ER, called the UPR. The role of the UPR is to sense ER protein-folding activities, signaling the genome to modulate the expression of genes affecting the protein folding machinery. In the co-splicing network presented in Fig. 6, the expression of 23 transcripts encoded by UPR-related genes was found to be correlated with *IREA1A*, *RBP-DR1*, and *At1g20880* transcripts, each encoding a cytoplasmic RBP (Additional file 15: Table S12).

**Fig. 7:**
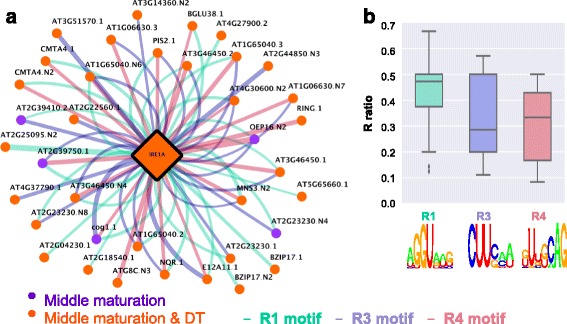
IRE1A co-splicing network **a** The co-splicing network for IRE1A illustrates the presence of consensus motifs in R1, R3, and R4 regions of potential pre-mRNAs targets. **b** R ratio distributions for IRE1A potential targets. R1, R3, and R4 motifs are present in more than 45, 30, and 32 % of the target exons.

**Fig. 8:**
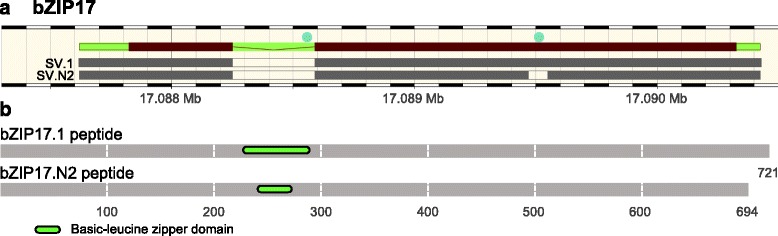
Gene structure and functional protein domains of bZIP17 isoforms. *bZIP17 SVs* were identified as potential products of IRE1A through co-expression and co-splicing network analyses. **a** *bZIP17* gene/pre-mRNA structure (exons are brown and introns are green) and the location of the predicted conserved motif in the R1 intron/exon splice junctions (teal circles) on the pre-mRNA. The predicted cryptic splice site is located in the middle of the second exon. **b** Protein isoforms encoded by the *bZIP17.1* and *bZIP17.N2* SVs with the location of a basic-leucine zipper domain. The interruption of the second exon with a short unconventional intron in *bZIP17.N2* results in the truncation of this domain.

The expression of a previously unknown IRE1A SV, an ER-resident transmembrane protein which carries out unconventional, extra-nuclear, splicing in the cytosol [36, 37], was found to be significantly correlated with the expression of 37 SVs (Additional file 16: Table S13, Fig. 7a). The motif present in R1 regions of these 37 SV is present in about 50 % of the exons, while R3 and R4 motifs are present in more than 30 % of the exons-intron junctions (Fig. 7b). All transcripts whose expression was correlated with that of *IRE1* fell into either the middle maturation or middle maturation and DT super-clusters. The expression of two SVs of bZIP17 TF, encoded by At2g40950, correlated with the expression of IRE1A, suggesting that *bZIP17* may be a product of IRE1A-associated splicing common to both SVs. Closer examination of *bZIP17* SVs and protein isoforms revealed the existence of a conserved (A/N)GGU(A/N)(A/T)(G/N) motif (Fig. 7) located in the R1 region of not only the first consensus intron, but also in a small and unconventional (cryptic) intron. An alternative donor and acceptor splice events within a large exon resulted in the formation of *bZIP17.N2* SV, which yielded a truncated protein due to truncation of the bZIP domain (Fig. 8). bZIP17 is a membrane-tethered, ER-localized TF activated by release from the ER membrane [38].

A search for common putative SF-binding motifs among the 45 SVs whose expression correlated with that of IRE1A revealed commonalities among R1, R3, and R4 groups. All detected R motifs were unique to the IRE1A group. This result suggests that additional direct targets of IRE1A may be present among this population of co-expressed and co-spliced transcripts. However, the expression of bZIP60, encoded by At1g42990, the most well established target of IRE1A for well-studied UPRs in seedlings, was not significantly correlated with the expression of IRE1A. Only the un-spliced form of bZIP60 was detected in developing Arabidopsis embryos, and it was significantly expressed during embryo development at the early maturation phase.

In addition, the expression of 15 transcripts encoded by genes associated with the UPR that were present in the early and middle maturation super-cluster was highly correlated with the expression of another SF, RBP-DR1, a cytoplasmic protein, encoded by At4g03110, which, to date has been associated only with the salicylic acid signaling pathway and responses to pathogens [40], and the regulation of flowering [41]. The inferred network for RBP-DR1 is available in Additional file 17: Table S14. Among the inferred targets of RBP-DR1 that are known to be UPR-related were a BIP, (At5g42020, an HSP 70 cognate), sec61 (At5g50460), a pre-protein translocase, (At3g60540), two SVs of PDI5, (At1g21750), PDI6, (At1g77510), and calnexin 1 (At5g61790). BIP is known to be a global regulator of the UPR [42].

The expression of a third cytoplasmic, relatively unstudied, RBP (At1g20880) was highly correlated with the expression of six UPR-related genes (At1g32560, At3g16990, At1g01580, a late embryogenesis related protein (LEA), a haem-oxygenase-like protein, and ferric reduction oxidase).

The presence of a motif in transcripts of several co-splicing groups would indicate that a motif is not specific for a particular SF. In order to examine whether motifs in the UPR-related co-splicing network involving IRE1A, RBP-DR1, and At1g20880 (Fig. 6) are specific or non-specific, the presence of the reported motifs was investigated in the rest of the co-splicing groups using FIMO search and the identified motifs were then subjected to the Chi-square test. The specificity test revealed that, in all cases, the motif was significantly specific (*p*-value <10E-6). Therefore, the motifs in all ER-related co-splicing groups are specific for each splice region.

Although the expression of other plant UPR-related genes/transcripts [38, 43] was not correlated with the expression of the three SFs discussed above, many UPR-related genes were significantly expressed in developing Arabidopsis embryos, including 17 transcripts that were present in the same super-clusters as the group of transcripts co-expressing with IRE1A. In addition, 49 other transcripts encoded by UPR-related genes were present in the early and middle maturation super-cluster (Additional file 18: Table S15).

## Discussion

### AS and protein diversity

AS-related diversity at the transcriptome level can result in an increase in protein plasticity in eukaryotes, as evident from proteomics data [44]. To address this question, a high-throughput RNA-Seq transcriptomics data set obtained from developing Arabidopsis embryos [21] was used to evaluate the changes in protein diversity caused by alternative splicing. This led to the identification of 9834 SV pairs encoded by 5602 genes in developing Arabidopsis embryos. Subsequent classification of transcripts into coding or noncoding using CodeWise [7] revealed that the majority of transcripts were predicted as coding and only 5.4 % of transcripts were noncoding, represented primarily by natural antisense and intergenic noncoding transcripts. Sense and antisense, and genic and intergenic long noncoding RNAs (lncRNAs) act in *cis* or *trans* as they can interact with nucleic acids or proteins and regulate gene expression through epigenetic, transcriptional, or post-transcriptional mechanisms [45–47].

Comparisons of sequences of *in silico* translated peptides revealed that approximately 24 % of the 9834 SV pairs had identical protein sequences. The same analysis was performed on the set of differentially expressed transcripts. Global pairwise alignment of protein isoforms in the latter population showed that AS did not change the open reading frame in the case of 34 % of the SV pairs. In these cases, some codons were removed during the splicing process. Approximately 40 % of the SV pairs showed amino-acid differences. Although these differences between protein isoforms make them different proteins, by definition, minor sequence differences may not result in a loss or gain of protein function and these peptides might be expected to have similar activities. AS at the unusual GYNGYN donor and NAGNAG acceptor splice sites at the exon/intron and intron/exon junctions, respectively, causes an insertion or deletion of three nucleotides without a frame shift in primary transcripts, resulting in a single amino acid change in the resulting proteins in eukaryotic organisms [48–50]. In some cases, insertion of three nucleotides may result in the introduction of a stop codon within the sequence of the final transcript, contributing to protein diversity [48, 49].

Loss, or truncation, of crucial functional domains, on the other hand, provide a potentially major contribution to protein diversity. Domain comparisons revealed that about 40 % of the peptide variant pairs detected contained identical functional domains. Approximately 23 % of the peptide variants had lost at least one functional domain and only 4 % had truncated domains. The remaining peptide variants did not have any known functional domains. The loss or truncation of at least one domain in 27 % of the peptide variants may affect their function, stability, regulation, and/or ability to interact with molecules. In terms of function-relevant protein diversity associated with AS, nearly a third of protein isoform pairs were considered to be diverse.

### Comparing CoSpliceNet with other existing methods

Emerging studies support the existence of co-splicing in plants [7, 20]. Pre-mRNA splicing is controlled by differentially expressed splicing regulatory proteins that confer splicing specificity in a cell-, development-, and/or growth condition-dependent manner [12]. Several co-splicing networks have recently been constructed to associate SFs with their target exons and introns and transcripts [51], for example, a Bayesian approach was used to generate a regulatory co-splicing network for the human SF Nova [17]. One of the goals in this rapidly growing field is to develop bioinformatics methods for inferring comprehensive co-splicing networks for a large number of SFs that would be suitable for cases where CLIP-based data are not available or limited, such as developing *Arabidopsis thaliana* embryos, the model system analyzed here.

We took advantage of an existing RNA-Seq dataset related to transcriptome changes during embryo development in Arabidopsis. To infer co-splicing networks and sub-networks in developing Arabidopsis embryos, functional associations between SFs and their products were predicted. This task was achieved by integrating transcript co-expression and splicing regulatory elements for 14 differentially expressed RBPs as *de novo* identified conserved multivalent RNA-binding motifs, to which SFs within spliceosomes are recruited for splicing.

Our method is distinct from other applications, such as RNAmotif, in several ways. First, RNAmotif combines R2 and R3 regions, whereas these exonic regions are separated in our method in order to be able to detect distinct RNA motifs at the 5′ and 3′ ends of exons. Second, we hypothesized that a SF and its potential product may be co-expressed. Therefore, the motif search was performed on co-expressing transcripts rather than on the entire population, enabling SF-specific motif and corresponding target transcript predictions. This also allowed the generation of co-splicing networks and sub-networks related to temporal aspects of embryo development in Arabidopsis using super-cluster information. Considering only differentially expressed SFs resulted in the elimination of SFs that were ubiquitously expressed and regulated at the post-translational levels. However, the goal of the current study was to identify developmentally related RBPs and their conserved RNA-binding motifs, which is the reason for selecting only differentially expressed RBPs. In many cases, the Ri ratio, which reflects how frequently a conserved motif was present in a transcript (*p*-value < 0.05 compared to the background noise), was less than 0.4, indicating that the motif was present in less than 40 % of the splice junctions. Therefore, some other SFs are likely involved in producing the final SVs.

Several motifs were specifically enriched in particular R regions. For example, the SR45-binding spliceosomal protein U2AF^35^ [30] was differentially expressed, and unique binding motifs were present in the R2 and R4 regions of the co-splicing group associated with U2AF^35^. Motifs in the R2 and R3 regions unique to the SC35 co-splicing group were also identified. SC35 is a member of the Ser/Arg-rich (SR) protein family, which are homologs of the corresponding SR protein family in mammals [31].

### ER-associated co-splicing sub-networks

The majority of transcripts are spliced in the nucleus concurrently with transcription of pre-mRNA. However, some pre-mRNAs are transported outside of the nucleus to the cytoplasmic side of the ER to get spliced [36]. Among the 13 RBPs within the co-splicing network, IRE1A, RBP-DR1, and At1g20880 were found to co-express with a number of transcripts involved in the UPR that are involved in maintaining proper protein folding at the ER. In addition, several enriched consensus RNA-binding motifs were identified on pre-mRNAs of these transcripts that may represent the specific binding sites for these three RBPs. RBP-DR1 and At1g20880 are cytoplasmic proteins with RNA-binding RRM/RBD/RNP motifs with no prior association with the ER-associated splicing of UPR-related targets [40, 54]. While the function of At1g20880 is unknown, RBP-DR1 is involved in promoting a hypersensitive response through positive regulation of salicylic acid signaling during plant-pathogen interactions and inhibition of flowering through mRNA decay of *SUPPRESSOR OF OVEREXPRESSION OF CONSTANS 1*, a component of flowering signaling pathway [40]. Based on a highly significant correlation between the expression of these RBPs and the expression of several UPR-related genes, RBP-DR1 and At1g20880 may be involved in UPR.

IRE1A is a well-studied, ER-localized transmembrane protein, which is documented to engage in unconventional, non-nuclear, splicing of pre-mRNAs present in the cytosol through the action of its C-terminal RNase domain facing the cytosol [39, 55]. Specifically, IRE1A is known to splice *bZIP60* pre-mRNA, encoding a TF that mediates the activation of some of the genes involved in the UPR in plants [56]. Two branches of the UPR are known in plants, one involving the splicing of bZIP60 and the other facilitating the release of two membrane-bound TFs in the ER, bZIP17 and bZIP28 [55]. Expression of two spliced forms of bZIP17 was correlated closely with the expression of IRE1A, suggesting that IRE1A is also involved in splicing *bZIP17* pre-mRNA. In contrast, only the unspliced form of bZIP60 was significantly differentially expressed in developing Arabidopsis embryos, suggesting that IRE1A did not act to splice the corresponding pre-mRNA. Based on these predictions and observations, the UPR in developing embryos may differ from that in seedlings in regard to the aspects related to *bZIP17* and *bZIP60* splicing.

IRE1A may have as yet unknown direct targets that modulate IRE1A signaling under specific conditions [57]. To date, the UPR has been studied almost exclusively in leaves and seedlings under different stress conditions. However, the role of IRE1A also appears to include effects on vegetative growth and reproductive development [56]. UPR-like events, called “ERQC, ER quality control”, can occur within the ER during development when high levels of secretory proteins are being synthesized, rather than a response to a classical stress condition [39]. This may also occur during the maturation and desiccation phases of Arabidopsis seed development when seed storage proteins are made. It should also be noted that, under normal conditions, loss of IRE1 causes changes in root growth [58], suggesting that this SF is also an essential part of development, in addition to its role in ER-related stress responses. Based on the transcriptomic data reported here, some form of this process is likely part of embryo development as 66 transcripts encoded by genes associated with ERQC were differentially expressed in developing Arabidopsis embryos, several of which were highly correlated with the expression of one or other of IRE1A or RBP-DR1. The splicing actions of IRE1A, RBP-DR1, and At1g20880 are likely to be part of this QC process. With respect to specific RNA-binding motifs, the AGGUAAG motif was found in the R1 region of the co-splicing group of IRE1A-associated transcripts (Fig. 7). This motif is similar to the binding motif of the RBP DAZAP1 (consensus motif UAGGUAG) [32] found in human reproductive tissues that is involved in both oocyte maturation [33] and spermatogenesis [34]. The UPR system has not previously been associated with the regulation of seed development, nor have specific SFs, nor have specific RNA-binding motifs been identified as part of that process. It is interesting to note that the three RBPs that showed high correlation with the expression of UPR-related transcripts are all cytoplasmic proteins. These observations suggest that the processing of this category of transcripts may have a cytoplasmic component and that non-nuclear splicing may be an essential part of the process of protein synthesis during seed development.

## Conclusions

Splicing is a highly regulated combinatorial process involving multiple SRPs and small nuclear RNA molecules interacting within the spliceosome and/or with pre-mRNA. CoSpliceNet has been developed for the inference of co-splicing networks through identification of SFs and their potential targets through joint analysis of co-expression and *de novo* motif prediction at the splice junctions. Pre-mRNA secondary and tertiary structures are known to be important for the regulation of splicing also [4, 52, 53]. Consequently, the integration of RNA structure and RNA-protein interactomes together with transcriptomic data is needed to obtain a comprehensive view of the regulation of splicing events [4]. The co-splicing networks presented here are predictive and intended to serve as a basis from which to begin to unravel this complex phenomenon. These networks facilitated the identification of groups of transcripts that were potential splice products of one or more SF. The pre-mRNAs of these candidate splice products possessed unique consensus *cis*-regulatory elements in at least one of their splice junctions, yielding predictions regarding the associations of SFs with their respective conserved RNA-binding motifs.

## Methods

### Glossary

Common terms used in this study are defined in Table 1.

### Transcriptomics data

The RNA-Seq data used for assessment of AS on protein diversity and inferring co-splicing networks were obtained from *Arabidopsis thaliana* embryos at the following stages: (i) early maturation (7 and 8 days after pollination (DAP)), (ii) middle maturation (10, 12 and 13 DAP), and (iii) early desiccation (15 and 17 DAP) phases [21]. At the early maturation stages, Arabidopsis embryos are already fully differentiated, and as they transition from torpedo to early bent cotyledon stage, they have already started accumulating seed storage compounds (oil and protein). Embryos at the middle maturation stage show steady-state accumulation of seed storage compounds [21] and as they begin to lose water during early desiccation stages, they start acquiring desiccation tolerance [22–24]. The accumulation of seed storage compounds and acquisition of desiccation tolerance prepares them for dormancy prior to germination. These phases of embryo development are characterized by specific metabolic, developmental, and signaling processes. 53,988 transcripts were detected in total, among which 7960 were identified as significantly differentially expressed when compared with the previous time point (*p*-value < 0.001 with fold change > 2).

### RNA-Seq analysis pipeline and the identification of differentially expressed transcripts

The RNA-Seq dataset (GEO accession number GSE74692) used in this report comprises seven time points, with three biological and four technical replicates per time point, representing different phases of Arabidopsis embryo development, from the onset of seed filling (7 days after pollination (DAP)) to the onset of seed desiccation (17 DAP). Read mapping, transcriptome assembly, and differential expression analyses were carried out using Tophat2 (version v2.0.13) [59], StringTie (version v1.0.1) [60], and Limma (version 3.3) [61] as described [21]. Briefly, the Arabidopsis reference genome (TAIR10 version) [54] was used to guide the transcriptome assembly process, yielding 41,933 known and 12,054 previously unreported expressed transcripts. Transcripts were defined as differentially expressed if their expression: (i) changed by at least 2 fold in a comparison of at least two time-points and (ii) was significantly different (*p*-value < 0.001) between any two consecutive time points. This analysis led to the detection of 7960 differentially expressed transcripts. This population was used for the study of SVs and their potential relationships with SRPs in co-expression and co-splicing networks. CodeWise [7] was used to assess the coding potential of transcripts. The expression of these 7960 transcripts was normalized using z-score and Cumulative Distribution Function (CDF) transformation. Subsequently, transcripts were clustered into 15, 20, 20, and 50 clusters. 50 clusters were finally chosen because they contained distinct expression patterns during embryo development (Additional file 3: Figure S2) to identify major expression trends using a k-means algorithm available in scikit-learn package [62], with the following parameters: init: ‘k-means++’, n_init = 1000, and max_iter = 1000. This setting stabilizes the k-means results, as each single run takes 1000 iterations, with 1000 initial different centroid seeds. This method was repeated for different number of clusters (15, 20, 20, and 50). Clusters containing transcripts that were expressed during the same phase of embryo development were subsequently merged into one of six resulting super-clusters.

### Characterization of transcripts

Categorization of the novel transcripts was performed according to the classes defined in the “Tuxedo Suite” package, which compares each assembled transcript with the closest transcript in the reference transcriptome and then assigns it to a class. These classes include j: novel transcript containing at least a novel splice junction, o: exon skipping, and x, s: antisense [7, 25, 26]. CodeWise [7] was used to classify all differentially expressed transcripts as coding or noncoding. CodeWise uses several features about the sequence and RNA secondary structure of transcript including conserved domains, ORF length, and RNA secondary structure free energy to identify noncoding and coding RNAs.

### Effects of AS on protein diversity

In order to assess the effects of AS on protein diversity, SVs encoding protein isoforms were compared with respect to differences in their overall sequence as well as in any conserved domains. A “canonical” transcript is defined as the SV with the lowest transcript number among known transcripts recorded in TAIR10 and was used as a reference for all comparisons. For example, At1g02850 has five known SVs. At1g02850.1 was used as the reference transcript, and all other SVs were compared to this canonical transcript. Note that the purpose of this section is to categorize the effect of AS on protein diversity, and therefore, the canonical transcript does not necessarily need to be differentially expressed.

Three parameters were assessed for this purpose: (i) peptide length ratio with respect to the canonical peptide, (ii) global pairwise alignment score, and (iii) conserved domain category. If gene X produces two SVs, SV1 and SV2, encoding the canonical protein isoforms X1 and the isoform X2 then the peptide length ratio will be defined as:

$$ \mathrm{Peptide}\kern0.5em \mathrm{length}\kern0.5em \mathrm{ratio}=\mathrm{length}\left(\mathrm{X}2\right)/\mathrm{length}\left(\mathrm{X}1\right) $$

Next, to identify sequence differences at the amino acid level, protein isoform X2 was aligned to the canonical protein isoform X1 using pairwise2 module available in biopython library with globalxx parameter. The globalxx parameter sets gap penalty to 0 and match score to 1. This setting makes the global alignment score to be the same as the number of matches. Therefore, if the alignment score is smaller than the length of the shorter peptide, at least a gap or mismatch exists between these protein isoforms. These results made the interpretation of the AS events straightforward in the context of relative protein sequence differences between two protein isoforms. The global alignment score was categorized into two groups based on the length of the non-canonical SV (X2): (i) alignment score = length(X2), suggesting that no mismatch exists between two protein isoforms (only gaps exist in pairwise global alignment) and (ii) alignment score < length(X2), suggesting presence of some mismatches in two protein isoforms.

Third, to predict how AS affects potential biological functions of protein isoforms, the corresponding protein isoforms were compared with respect to their conserved functional domains. Batch Conserved Domain Search (CD-Search) [63] with the default setting was used to identify conserved domains in protein isoforms. Non-specific hits were subsequently filtered from the CD-Search outputs, leaving only superfamily and specific hits. Protein isoforms were categorized into four groups based on differences in their domains: (i) disparate domains – found in protein isoforms that had different number of conserved domains, (ii) identical domains – found in protein isoforms that had the same number of functional domains, (iii) similar domains – found in isoforms that had the same number of conserved domains, but at least one domain was truncated in one of the isoforms, and (iv) no domains – relevant to isoforms with no known domains (found predominantly in proteins of unknown function).

### CoSpliceNet - Co-splicing network construction

The bioinformatic pipeline for constructing co-splicing networks from transcriptomics data is presented in Fig. 4. The detailed step-by-step CoSpliceNet framework is available in Additional file 19: Figure S4.

### Identification of 146 differentially expressed SRPs and 14 RBPs containing RNA-binding domains

A list of genes encoding SRPs in Arabidopsis was obtained by combining entries from the Arabidopsis Splicing-Related Genes (ASRG) database (395 SRPs) [64] and the results of a proteomics analysis performed on isolated Arabidopsis spliceosomes (additional 89 SRPs) [65]. An additional 14 SRPs were identified from the literature, yielding a total of 497 SRPs. The list of 497 SRPs was compared to the list of 7960 differentially expressed transcripts, and 146 differentially expressed SRPs were identified. Among these 146 SRPs, 14 were identified as RBPs that contained at least one RNA-binding domain and could represent SFs.

### Co-expression network construction

Correlation analysis was performed to identify transcripts whose expression patterns were highly correlated with at least one of the 146 differentially expressed SRPs (*p*-value < 0.001, Spearman correlation coefficient > 0.95). The SRPs and their correlated transcripts were visualized as a co-expression network in Cytoscape [66] using the organic layout. All subsequent networks and sub-networks were visualized this way.

### *De novo* RNA-binding motif discovery and co-splicing network construction

Several steps were performed for each RBP to construct a co-splicing network (Fig. 1). First, transcripts whose expression was highly correlated with the expression of at least one of the 14 RBPs were identified. *De novo* motif discovery was then performed using MEME [28] to identify consensus sequences in 30-nucleotide (R1 – 4) R-regions, near splice sites for each exon/intron junction of co-expressed transcripts. R1 and R4 are within intronic, while R2 and R3 are within exonic regions (Fig. 4). Thirty-nucleotide R regions were extracted from upstream (minus signs) and downstream (plus signs) of exon/intron junctions (5′- and 3′-splicing sites “ss”) as follows: R1 (-31:-1 5′ss), R2 (0:30 5′ss), R3 (-30:0 3′ss), and R4 (1:31 3′ss) sequences for each exon in a transcript, yielding four sets of sequences for each intron-exon-intron region (SF_Ri.fasta, 1 ≤ i ≤ 4) as suggested in RNAmotif tool [19]. The choice of this search window length is also supported by recent published data showing the presence of binding site enrichment in close proximity of splicing sites in either upstream or downstream sequences and similar lengths have been used by other tools [19, 20, 29]. We refer to an R region as Ri (1 ≤ i ≤ 4). Third, each SF had a separate SF_Ri.fasta containing these 30-nucleotide exonic and intronic sequences from co-expressing transcripts.

Due to the lack of available CLIP data for developing Arabidopsis embryos, *de novo* motif discovery was performed on sequences in 14 SF_Ri.fasta files using MEME [28] in ZOOPS mode (Zero or One Occurrence Per Sequence) to identify the top consensus k-mer motif (4 ≤ k ≤ 7) in each R region (motif E-value < 0.01). Transcripts with a significant motif located in at least one of their R regions with *p*-value < 0.05 (compared with the background noise) were identified. We hypothesized that if an exon or intron contains a significant motif in a Ri region, then the co-expressing SF is involved in splicing that particular transcript through binding to that RNA motif. A transcript has generally more than one exon and, therefore, a conserved motif could potentially be present in multiple Ri regions. In order to investigate how frequent a motif is found in each Ri region, a metric called Ri ratio was defined for each transcript. Ri ratio was calculated as the fraction of exons containing a motif in their Ri region to total number of exons in a transcript.

An edge (corresponding to a Ri) was formed between a SF and each predicted product transcript in the co-splicing network if at least one of the exons or introns in the co-expressed transcript contained a significant conserved motif in the Ri region. Therefore, a SF can be connected to a potential target via at most four edges corresponding to the presence of a significant motif in each Ri regulatory region. Motifs were compared with published RNA-binding motifs using TOMTOM tool, which is also available in the MEME suite [67].

## Additional files

Additional file 1: Figure S1. Protein diversity analysis of the set of differentially expressed transcripts expressed during Arabidopsis embryo development. Two thousand three hundred forty-five genes were alternatively spliced. Protein diversity analysis was performed on 3008 SV pairs of these genes. a Effect of coding potential on peptide length differences of protein isoforms. b Relationship between the domain composition and coding potential. c Relationship between the peptide length ratio and coding potential. (PDF 130 kb)
Additional file 2: Table S1. The set of 7960 differentially expressed transcripts in developing Arabidopsis embryos. Data related to expression values, clusters, coding potential, domains, and TAIR10 functional description are provided for each transcript. (XLSX 3712 kb)
Additional file 3: Figure S2. The k-means clusters. The set of 7960 differentially expressed transcripts was clustered into 50 clusters using k-means algorithm. (PDF 1583 kb)
Additional file 4: Table S2. List of 146 differentially expressed SRPs and their functional and other properties. (XLSX 86 kb)
Additional file 5: Figure S3. Distribution of the 146 SRPs in the super-clusters (PDF 124 kb)
Additional file 6: Table S3. List of transcripts and their properties associated with each SRP in the eye-shaped co-expression network. (XLSX 585 kb)
Additional file 7: Table S4. List of 14 differentially expressed RBP transcripts. (XLSX 28 kb)
Additional file 8: Table S5. RBP-specific co-expression network. Association of 2646 differentially expressed transcripts that were co-expressed with 14 RBPs. (XLSX 511 kb)
Additional file 9: Table S6. Co-splicing network of 2332 transcripts associated with 13 RBPs. (XLSX 1313 kb)
Additional file 10: Table S7. Significant associations between RBPs and their potential products. The R regions that do not have a significant motif were filtered using *p*-value cutoff of 0.05. The log(*p*-value) was used to weight the edges in the co-splicing network. (XLSX 213 kb)
Additional file 11: Table S8. Enriched motifs in R regions of targets of each RBP. The motifs are shown as logos from the MEME analysis. (XLSX 2349 kb)
Additional file 12: Table S9. Enriched motifs in R regions of targets of each RBP. The motifs are shown as regular expressions from the MEME analysis. (XLSX 50 kb)
Additional file 13: Table S10. Position weight matrices of the motifs enriched in R regions of targets of each RBP. (TXT 22 kb)
Additional file 14: Table S11. Differentially spliced transcripts. (XLSX 65 kb)
Additional file 15: Table S12. ER-related transcripts in the UPR-related co-splicing network. (XLSX 55 kb)
Additional file 16: Table S13. IRE1A co-splicing network. (XLSX 82 kb)
Additional file 17: Table S14. RBP-DR1 co-splicing network. (XLSX 134 kb)
Additional file 18: Table S15. ER-related transcripts identified in the set of 7960 differentially expressed transcripts. (XLSX 89 kb)
Additional file 19: Figure S4. Step-by-step bioinformatics pipeline for co-splicing network construction from expression data (PDF 102 kb)

## Abbreviations

AS: Alternative splicing
ASRG: Arabidopsis Splicing-Related Genes
CDF: Cumulative distribution function
CD-Search: Conserved Domain Search
co-splicing: Coordinated splicing
DAP: Days after pollination
ER: Endoplasmic reticulum
LEA: Late embryogenesis related protein
lncRNA: Long noncoding RNA
ncRNA: Noncoding RNA
PAR-CLIP: Photoactivatable Ribonucleoside-Enhanced Crosslinking and Immunoprecipitation
pre-mRNA: Precursor mRNA
RBP: RNA-binding protein
RNA-Seq: RNA sequencing
SF: Splicing factor
SRP: Splicing-related proteins
SV: Splice variant
UPR: Unfolded protein response
ZOOPS: Zero or One Occurrence Per Sequence
