# Supplementary material for: CoSpliceNet: a framework for co-splicing network inference from transcriptomics data
Source: BMC Genomics. 2016 Oct 28;17:845. doi: 10.1186/s12864-016-3172-6 (PMC5086072; doi:10.1186/s12864-016-3172-6)
Supplement: Additional file 1: Figure S1. — Protein diversity analysis of the set of differentially expressed transcripts expressed during Arabidopsis embryo development. Two thousand three hundred forty-five genes were alternatively spliced. Protein diversity analysis was performed on 3008 SV pairs of these genes. a Effect of coding potential on peptide length differences of protein isoforms. b Relationship between the domain composition and coding potential. c Relationship between the peptide length ratio and coding potential. (PDF 130 kb) [file 12864_2016_3172_MOESM1_ESM.pdf]

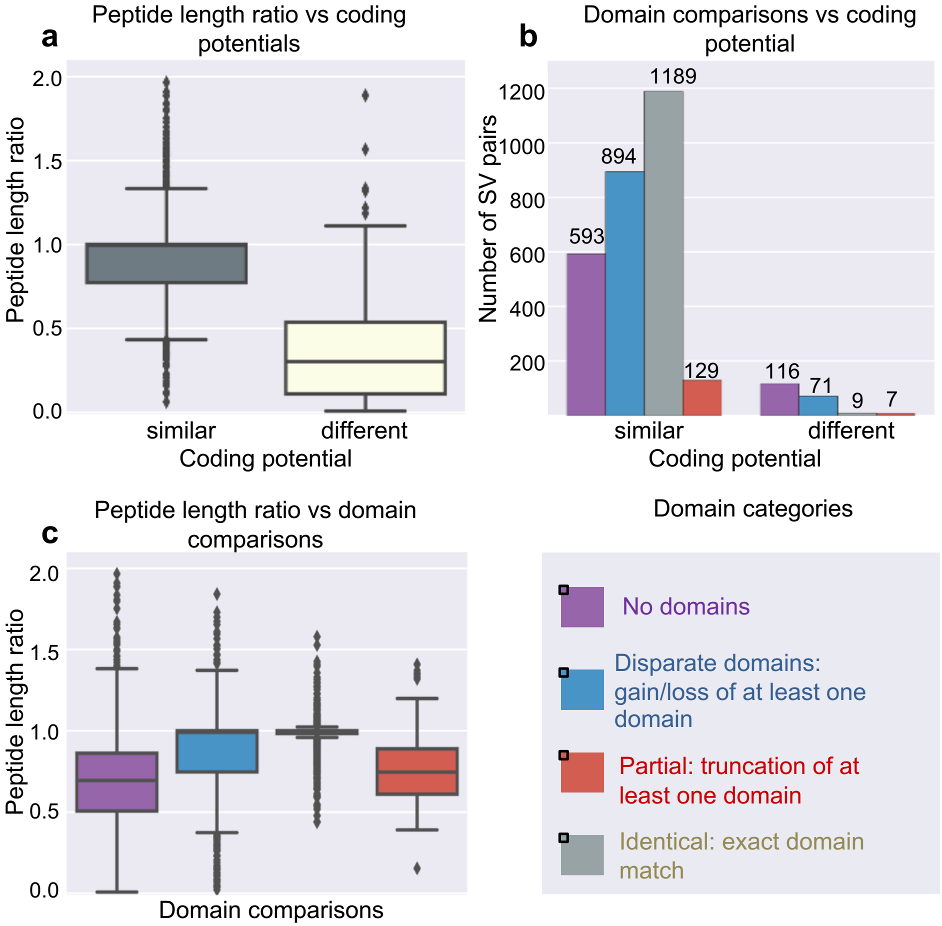

Additional File 1: **Figure S1.** Protein diversity analysis on the set of differentially expressed transcripts during Arabidopsis embryo development. 2,345 genes were alternatively spliced. Protein diversity analysis was performed on 3,008 SV-pairs of these genes. a Effect of coding potential on peptide length differences of protein isoforms. b Relationship between the domain composition and coding potential. c Relationship between the peptide length ratio and coding potential.
