# Supplementary material for: CoSpliceNet: a framework for co-splicing network inference from transcriptomics data
Source: BMC Genomics. 2016 Oct 28;17:845. doi: 10.1186/s12864-016-3172-6 (PMC5086072; doi:10.1186/s12864-016-3172-6)
Supplement: Additional file 3: Figure S2. — The k-means clusters. The set of 7960 differentially expressed transcripts was clustered into 50 clusters using k-means algorithm. (PDF 1583 kb) [file 12864_2016_3172_MOESM3_ESM.pdf]

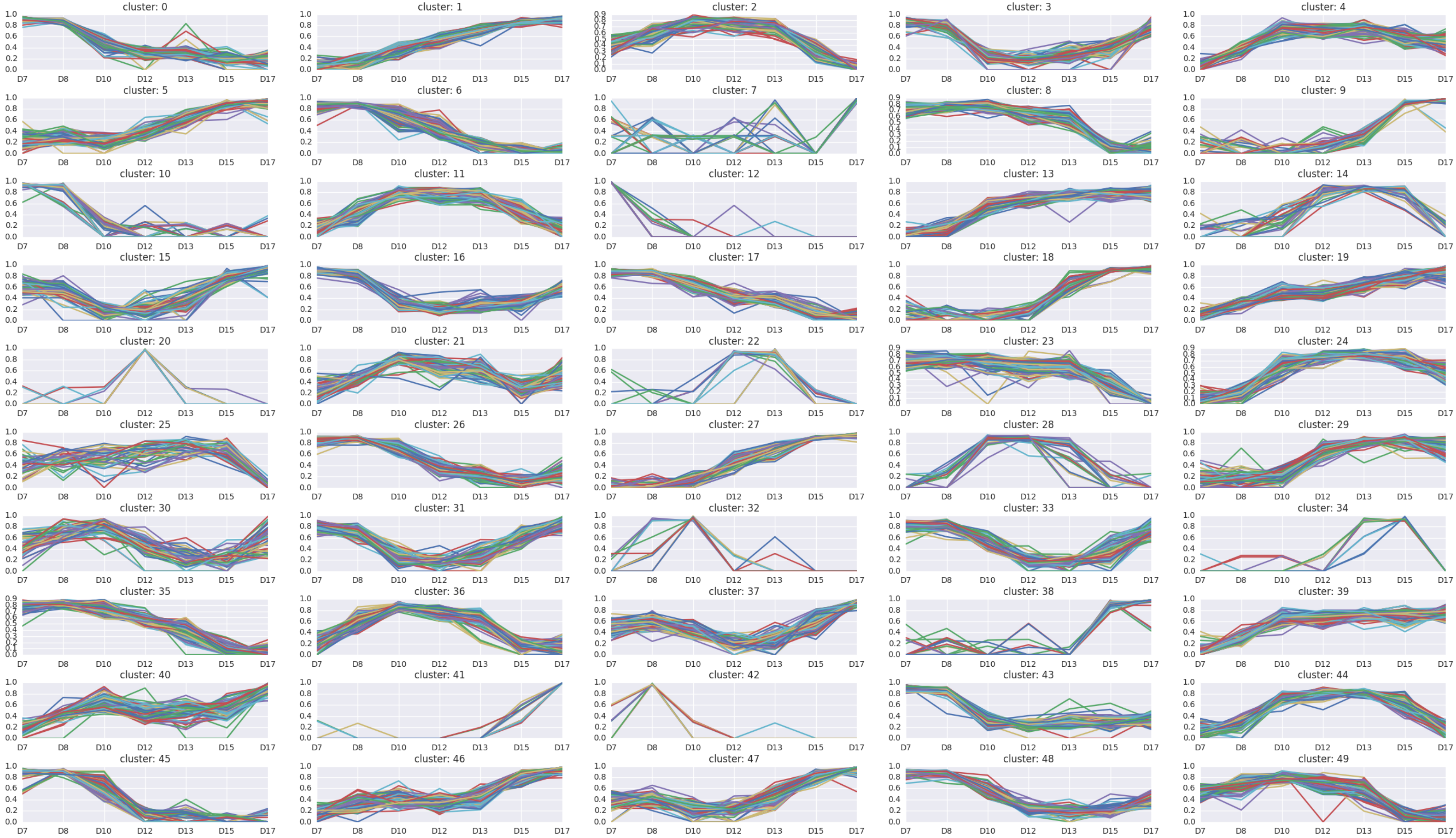

Additional File 3: **Figure S2.** The set of 7,960 differentially expressed transcripts was clustered into 50 clusters using K-means algorithm.
