# Supplementary figures and images for: CoSpliceNet: a framework for co-splicing network inference from transcriptomics data
Source: BMC Genomics. 2016 Oct 28;17:845. doi: 10.1186/s12864-016-3172-6 (PMC5086072; doi:10.1186/s12864-016-3172-6)

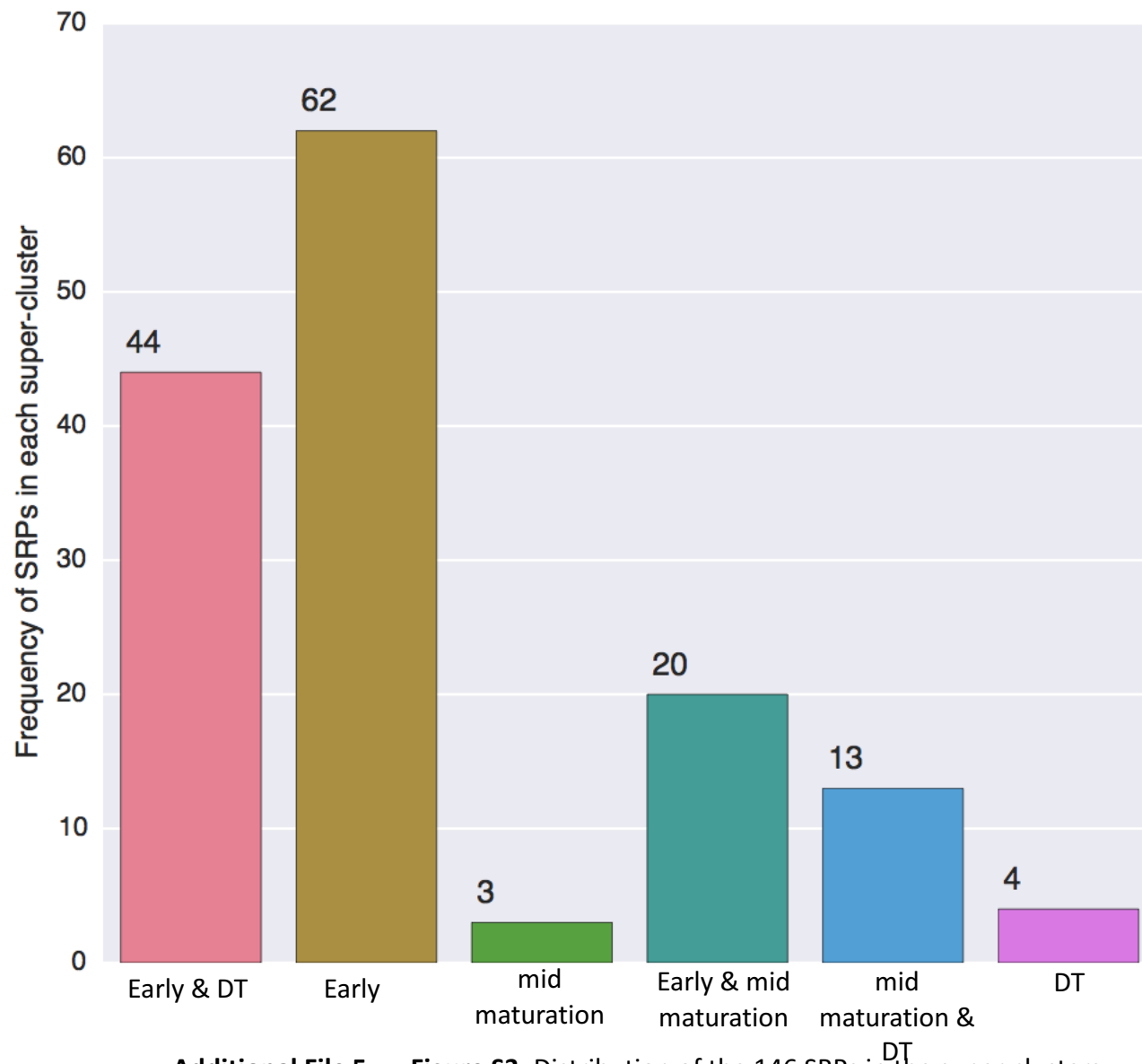

**Additional File 5: Figure S3.** Distribution of the 146 SRPs in the super-clusters

Supplement: Additional file 5: Figure S3. — Distribution of the 146 SRPs in the super-clusters (PDF 124 kb) [file 12864_2016_3172_MOESM5_ESM.pdf]
