# Supplementary material for: CoSpliceNet: a framework for co-splicing network inference from transcriptomics data
Source: BMC Genomics. 2016 Oct 28;17:845. doi: 10.1186/s12864-016-3172-6 (PMC5086072; doi:10.1186/s12864-016-3172-6)
Supplement: Additional file 19: Figure S4. — Step-by-step bioinformatics pipeline for co-splicing network construction from expression data (PDF 102 kb) [file 12864_2016_3172_MOESM19_ESM.pdf]

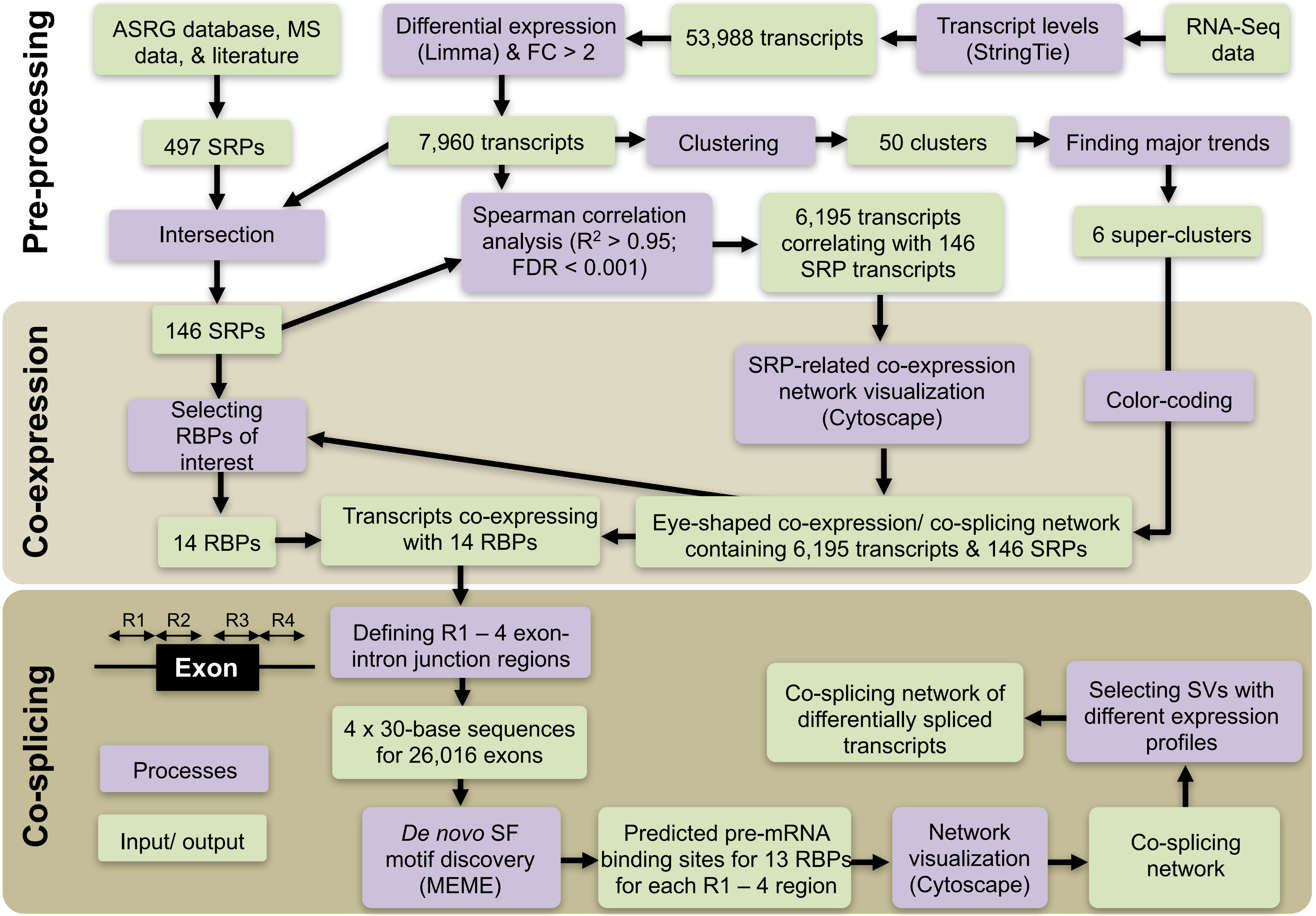

Additional File 19: Figure S4. Step-by-step bioinformatics pipeline for co-splicing network construction from expression data
